# Multimodal analysis of granulocytes, monocytes, and platelets in patients with cystic fibrosis before and after Elexacaftor–Tezacaftor–Ivacaftor treatment

**DOI:** 10.3389/fimmu.2023.1180282

**Published:** 2023-06-29

**Authors:** Hanna Schmidt, Larissa Melina Höpfer, Lisa Wohlgemuth, Christiane Leonie Knapp, Adam Omar Khalaf Mohamed, Laura Stukan, Frederik Münnich, Dominik Hüsken, Alexander Sebastian Koller, Alexander Elias Paul Stratmann, Paul Müller, Christian Karl Braun, Dorit Fabricius, Sebastian Felix Nepomuk Bode, Markus Huber-Lang, David Alexander Christian Messerer

**Affiliations:** ^1^ Department of Pediatric and Adolescent Medicine, University Hospital Ulm, Ulm, Germany; ^2^ Institute of Clinical and Experimental Trauma Immunology, University Hospital Ulm, Ulm, Germany; ^3^ Institute of Transfusion Medicine, Ulm University, Ulm, Germany; ^4^ Institute of Clinical Transfusion Medicine and Immunogenetics Ulm, German Red Cross Blood Transfusion Service and University Hospital Ulm, Ulm, Germany; ^5^ Department of Transfusion Medicine and Hemostaseology, Friedrich-Alexander University Erlangen-Nuremberg, University Hospital Erlangen, Erlangen, Germany

**Keywords:** cystic fibrosis, neutrophils, monocytes, Elexacaftor–Tezacaftor–Ivacaftor, cystic fibrosis transmembrane conductance regulator, CFTR modulator therapy

## Abstract

Cystic fibrosis (CF) is a monogenetic disease caused by an impairment of the cystic fibrosis transmembrane conductance regulator (CFTR). CF affects multiple organs and is associated with acute and chronic inflammation. In 2020, Elexacaftor–Tezacaftor–Ivacaftor (ETI) was approved to enhance and restore the remaining CFTR functionality. This study investigates cellular innate immunity, with a focus on neutrophil activation and phenotype, comparing healthy volunteers with patients with CF before (T1, n = 13) and after six months (T2, n = 11) of ETI treatment. ETI treatment reduced sweat chloride (T1: 95 mmol/l (83|108) vs. T2: 32 mmol/l (25|62), p < 0.01, median, first|third quartile) and significantly improved pulmonal function (FEV_1_ T1: 2.66 l (1.92|3.04) vs. T2: 3.69 l (3.00|4.03), p < 0.01). Moreover, there was a significant decrease in the biomarker human epididymis protein 4 (T1: 6.2 ng/ml (4.6|6.3) vs. T2: 3.0 ng/ml (2.2|3.7), p < 0.01) and a small but significant decrease in matrix metallopeptidase 9 (T1: 45.5 ng/ml (32.5|140.1) vs. T2: 28.2 ng/ml (18.2|33.6), p < 0.05). Neutrophil phenotype (CD10, CD11b, CD62L, and CD66b) and function (radical oxygen species generation, chemotactic and phagocytic activity) remained largely unaffected by ETI treatment. Likewise, monocyte phenotype and markers of platelet activation were similar at T1 and T2. In summary, the present study confirmed a positive impact on patients with CF after ETI treatment. However, neither beneficial nor harmful effects of ETI treatment on cellular innate immunity could be detected, possibly due to the study population consisting of patients with well-controlled CF.

## 1 Introduction

Cystic fibrosis (CF) is one of the most common life-threatening autosomal-recessive monogenetic diseases affecting over 100 000 people globally and is caused by mutations in the gene that codes for the cystic fibrosis transmembrane conductance regulator (CFTR) (1–3). CFTR is an epithelial ion channel that transports chloride and bicarbonate across the apical surface of secretory epithelia (1, 4). Therefore, CF is a multi-organ pathology that alters mucus secretion in the upper and lower airways, the gastrointestinal tract that includes the pancreas, and the endocrine and reproductive systems (1, 2, 4, 5). Currently, over 2000 different mutations have been described, which are summarized in six classes (2). However, in approximately 85% of patients with CF, at least one allele of the CFTR gene is affected by the most common mutation c.1521_1523del, resulting in the deletion of p.Phe508 (NM_000492.3: c.1521_1523del, hereafter referred to as p.Phe508del, dbSNP: rs113993960). This causes defective intracellular processing, impaired trafficking, and decreased protein stability, subsequently reducing the levels of intact CFTR protein on the apical surface of epithelial cells (1, 3, 4, 6, 7).

While the initial treatment focused on symptomatic intervention, for example, by assisting expectoration, nutritional supplementation, and antibiotic treatment of chronic and/or exacerbated infections, modern treatments also aim to directly restore CFTR function (1, 4). Depending on the individual mutations, these CFTR modulators partially restore CFTR defects improving clinical outcome in patients with CF (1, 4). The first CFTR modulator (Ivacaftor (IVA)) was approved by the European Medicines Agency (EMA) and the US-American Federal Drug Agency (FDA) in 2012. Although many patients with CF experienced a benefit by IVA therapy (or subsequently developed combinations of IVA and a second modulator, Tezacaftor (TEZ)), there were no sufficient treatment options for approximately 30% of the patients with CF. This group included patients with CF who are heterozygous for p.Phe508del and a mutation of minimal function (defined as a mutation that does not produce protein or produces protein that is resistant to IVA, TEZ, or the combination of IVA–TEZ) (1, 4, 8).

To address this hitherto unmet clinical need, a triple combination of CFTR modulators (Elexacaftor–Tezacaftor–Ivacaftor, tradename EU: Kaftrio, tradename USA: Trikafta, hereafter referred to as ETI) was developed (4). The next-generation corrector Elexacaftor improves CFTR protein processing and trafficking via a mechanism different from that of the first-generation corrector TEZ. The potentiator IVA increases CFTR channel open probability. In vitro, the ETI combination restored CFTR function more effectively than its single components (9). Phase 2 and 3 clinical trials confirmed substantial beneficial effects on clinical endpoints, including the forced expiratory volume in one second (FEV_1_), pulmonary exacerbations, sweat chloride concentration, and body mass index (BMI = kg/m²) (4, 9). ETI was first approved by the FDA and the EMA in 2019 and 2020, respectively (10). Currently, ETI is licensed by the EMA for the treatment of patients aged from 6 years with CF with at least one p.Phe508del mutation (11).

Sustained inflammation plays a critical role in CF lung disease, which is predominantly neutrophil driven but also promoted by monocytes and platelets (12, 13). Recurrent lung infections and infectious exacerbations contribute relevantly to disease progression (1, 12). Because neutrophils provide the first line of cellular defense in bacterial lung infections, proper neutrophil function, particularly in the context of CF, is crucial for the clearing of bacteria and resolving inflammation (12, 14). However, functional investigation of neutrophils and monocytes as the vanguard of innate immunity in CF revealed cellular dysfunction, including impaired ability to kill phagocytosed bacteria (5, 15), alterations in migration and chemotaxis (16, 17), and delayed apoptosis (18). The described defects in innate immunity presumably contribute to the failure to clear bacterial infections despite high levels of neutrophil recruitment (12, 18). In general, the neutrophil count was reported to increase in patients with CF, but decreased after ETI treatment (19). Additionally, the neutrophil phenotype in patients with CF was similar to that of healthy volunteers, but changed during infectious exacerbation (20).

Neutrophils, monocytes, and platelets can become activated by a variety of mediators of inflammation, for example, cytokines such as tumor necrosis factor (TNF), lipid-derived mediators such as platelet-activating factor (PAF), and microbe-associated molecular patterns (MAMPs, e.g., N-formylmethionyl-leucyl-phenylalanine (fMLF) or lipopolysaccharide (LPS)), and others (21–23). Upon activation, neutrophils respond with a defined response in changes of cellular physiology such as the intracellular pH and alterations in markers of cellular activation (21, 22, 24, 25). The latter include the expression of CD11b and CD62L on neutrophils and monocytes as well as CD42b and CD62P on platelets, respectively (22, 26, 27). Besides their involvement in cellular activity such as extravasation or the formation of platelet-neutrophil complexes (PNCs) or platelet-monocyte complexes (PMCs), respectively, these activation markers are also used as surrogates to monitor infection related inflammation in general as well as in the context of CF (20, 24–26). For example, patients with CF responded with a more pronounced CD11b upregulation upon stimulation with fMLF in comparison to healthy subjects (26).

In summary, it remains a matter for debate whether dysregulation of innate immunity in CF is acquired or constitutive (28) and whether CFTR modulator therapy directly affects cellular innate immunity (29, 30). Therefore, the present study investigated the phenotype and cellular function of neutrophils and monocytes under resting conditions and after their exposure to inflammatory mediators, the cells being from patients with CF before and after ETI treatment compared to healthy volunteers.

## 2 Methods

### 2.1 Study cohort, blood sampling, and clinical data

**Figure 1:**
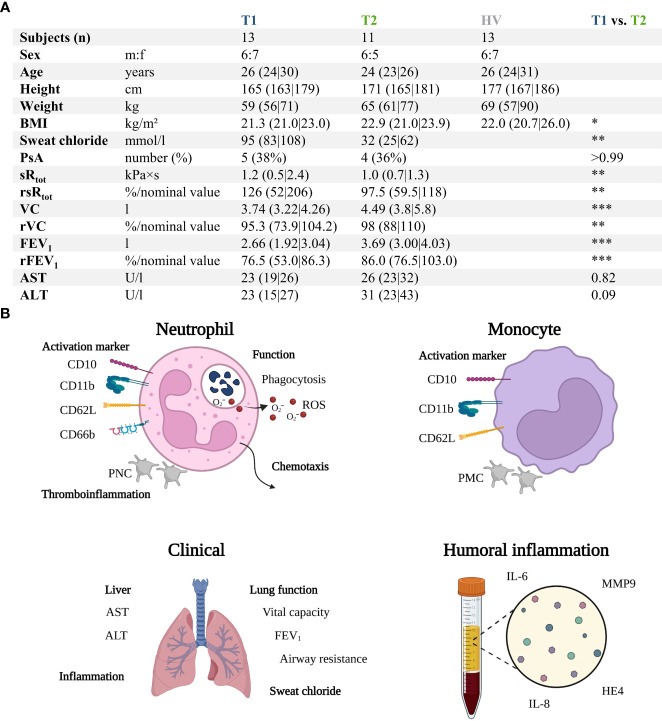
Summary of the prospective observatory study. **(A)** Patients with CF prior to treatment (T1) with Elexacaftor–Tezacaftor–Ivacaftor (ETI) were compared to age- and sex-matched healthy volunteers (HV) as well as after 6 months of ETI treatment (T2). **(B)** Graphical synopsis of the parameters analyzed. ALT , alanine aminotransferase; AST , aspartate aminotransferase; BMI , body mass index; FEV_1_ , forced expiratory volume in one second; HE4 , human epididymis protein 4; IL-6 , interleukin 6; IL-8 , interleukin 8; MMP9 , matrix metallopeptidase 9; PNC , platelet-neutrophil complex; PsA , positive for Pseudomonas aeruginosa within the previous 6 months; rFEV_1_ , relative forced expiratory volume in one second; ROS , radical oxygen species; rsR_tot_ , relative total specific airway resistance; rVC , relative vital capacity; sR_tot_ , total specific airway resistance; VC , vital capacity. *, **, ***, denote p < 0.05, 0.01, and 0.001, respectively.

All experiments were performed in accordance with the Helsinki declaration (31), after ethical approval (number 327/20, Local Independent Ethics Committee of the University of Ulm), and after obtaining written informed consent. The study included patients with previously diagnosed CF as well as age- (± 1 year) and sex-matched healthy volunteers (HV) as summarized in **Figure 1**. CF patients were analyzed prior to the initiation of treatment (T1) and during a follow-up visit after 6 months ((T2), median 6 months (6.0|6.5)). Patients were screened for eligibility to receive ETI treatment (either as a first CF-specific treatment or as a change in treatment regimen) during routine visits to the outpatient clinic of the Department of Pediatrics and Adolescent Medicine, University Medical Center Ulm. Inclusion criteria were (I) age > 18 years and (II) homozygous p.Phe508del mutation or compound heterozygous p.Phe508del mutation (in accordance with the approved indications for ETI). Exclusion criteria were (I) acute infection (II), fever or invasive procedures during the previous seven days (III), immunosuppressive medication, and (IV) systemic antimicrobial therapy during the three days prior to blood sampling.

Blood was drawn by peripheral venipuncture in adherence to the guidelines of the World Health Organization (32) and collected in monovettes containing 3.2% trisodium citrate (Sarstedt, Nümbrecht, Germany), 35 IU/ml Heparin (Sarstedt), or 1.6 mg/ml K3 EDTA (Sarstedt). During the respective consultation in the outpatient clinic, routine clinical data was obtained and analyzed including height, weight, BMI, chloride concentration of sweat collected via pilocarpine iontophoresis (Macroduct Sweat collector Webster Modell 3700, Wesco, Logan, USA), and lung function (MasterScreen Body, Vyaire Medical GmbH, Höchberg, Germany). Aspartate transaminase (AST) and alanine transaminase (ALT) were determined by photometric analysis using the Cobas c system (photometric measurement, Roche, Basel, Switzerland), and the differential blood count was obtained using a standard hematology analyzer (Sysmex CN 2000, Sysmex, Kobe, Japan). To estimate the microbial burden of the patients, sputum (or in case of non-expectorating patients: throat swaps) was collected during regular visits for microbial analysis. To reduce false-negative findings, the results of two independent samples were included when available (T1: approximately 3 months prior to the initiation of treatment and at the initiation of the treatment; T2: 6 months after the initiation of the treatment and in a follow-up visit approximately 9 months after the initiation of the treatment). If one of the two samples for the respective measurement point became positive, the patient was considered positive for the respective microbial agent. It should be noted that the microbial data set should be interpreted with caution, because the distribution of sputum and throat swaps changed after ETI treatment (T1: 84% vs. T2: 43% sputum).

For stimulation and subsequent staining, 10 µl citrate-anticoagulated blood were added to PBS^++^ (Dulbecco’s Phosphate Buffered Saline including calcium and magnesium, #14040-091, Gibco Thermo Fisher Scientific, Darmstadt, Germany) adjusted to pH 7.3. The total volume of blood and PBS including stimuli and staining reagents cumulated to 50 µL. Blood was stimulated with PBS as buffer control, 1 µM PAF (PAF C-18:1, #85966-90-1, Cayman Chemical Company, Ann Arbor, USA), 100 ng/ml LPS from Escherichia coli (hereafter referred to as LPS EC, Escherichia coli O55:B5, #L2880, Sigma Aldrich, Steinheim, Germany), 1 µg/mL LPS from Pseudomonas aeruginosa (hereafter refered to as LPS PsA, Pseudomonas aeruginosa 10, #L8643, Sigma Aldrich), or a mixture of inflammatory mediators (hereafter referred to as the mixture of proinflammatory mediators or Cocktail in the figures) consisting of 1 µM PAF, 10 µM fMLF (#F3506, Sigma Aldrich), and 2.3 µM TNF (#570104, BioLegend, San Diego, USA) as indicated in the figure captions. Subsequently, the cells were stained, chemically fixed, and measured as described below. PAF, LPS, fMLF, and TNF were chosen as commonly used and clinically relevant stimuli of cellular innate immunity (21, 22, 26, 27). Stimulation only by PAF and the stimulation with the mixture of proinflammatory mediators was chosen to elicit a medium and a strong inflammatory response based on unpublished preliminary results and as confirmed in **Figure 3**. LPS from Pseudomonas aeruginosa and Escherichia coli was used to briefly simulate exposure to pathogens.

### 2.2 ELISA

Citrate anticoagulated blood was centrifuged for 10 minutes at 400 × g. The sampled plasma was stored at −80°C until further use for the analysis of humoral markers of inflammation. Measurement of plasma levels of interleukin 6 (BD OptEIA Human IL-6 ELISA Set, #555220, BD Biosciences, San Jose, USA), interleukin 8 (DuoSet^®^ Human IL-8/CXCL8 ELISA Kit, #DY208, R&D Systems, Minneapolis, USA), matrix metallopeptidase 9 (DuoSet^®^ Human MMP9 ELISA Kit, #DY911, R&D Systems), and human epididymal protein 4 (also known as WAP four-disulfide core domain protein 2 (WFDC2), DuoSet^®^ Human HE4/WFDC2 ELISA Kit, #DY6274-05, R&D Systems) was carried out using standard enzyme-linked immunosorbent assays (ELISAs) as indicated by the manufacturers.

### 2.3 Flow cytometry analysis of neutrophils and monocytes

For the analysis of the neutrophil and monocyte phenotype as previously described (21, 22), 10 µl citrate-anticoagulated blood were added to 40 µl PBS^++^ adjusted to pH 7.3 including prior added stimuli and antibodies as listed below and incubated for 15 minutes in a light-protected water bath at 37°C. The diluted whole blood was stained as indicated with anti-CD10 (PE-Cyanine7 anti-human CD10, dilution 1:1666.7, #312214, BioLegend), anti-CD11b (APC anti-mouse/human CD11b, dilution 1:3333, #101212, BioLegend), anti-CD62L (PE anti-human CD62L, dilution 1:400, #304806, BioLegend), anti-CD66b (APC-Cyanine7 anti-human CD66b, dilution 1:200, #305126, BioLegend), or corresponding isotype controls (all from BioLegend). In addition, the diluted whole blood was stimulated with either PBS^++^ (as buffer control, hereafter referred to as Ctrl), PAF, LPS or with the mixture of proinflammatory mediators described above.

Similarly, to assess neutrophil activity, 10 µl heparin-anticoagulated blood were added to 40 µl PBS^++^ adjusted to pH 7.3 (including prior added stimuli as listed above and fluorescent reagents as subsequently listed) and incubated for 30 minutes at 37°C in a light-protected water bath. Phagocytosis was analyzed using fluorescent microspheres (Fluoresbrite BB Carboxylate 0.50 Micron Microspheres, Polysciences, Inc., Warrington, USA). The microspheres were dissolved 1:10 in PBS^++^ followed by a washing procedure (3 × at 4000 × g for 5 minutes). Of this microsphere solution, 5 µl was added to the above-mentioned mixture resulting in a total volume of 50 µl. Radical oxygen species (ROS) generation was determined by adding 5 µM CellROX Deep Red (#C10422, Thermo Fisher Scientific). Following stimulation and staining of diluted whole blood as described above, erythrocytes were lysed and leukocytes fixed in a sample volume made up to 1 mL with 1 × BD FACS lysing solution (#349202, BD Biosciences) for 30 minutes and incubated at room temperature in the dark. Following centrifugation of the samples for 5 minutes at 340 × g, the pellet was resuspended in 100 µl PBS^++^ containing 0.1% bovine serum albumin (Sigma Aldrich) and stored at room temperature in the dark until further analysis.

To briefly analyze changes in neutrophil cellular physiology, the membrane potential (MP) and intracellular pH (pH_i_) was monitored by using fluorescent dyes as described before (33–36) with brief modifications as subsequently described. 5µl citrate anticoagulated blood was mixed with 40 µl PBS^−−^ (Dulbecco’s Phosphate Buffered Saline, #14190-094, Gibco Thermo Fisher Scientific) including anti-CD45 (Pacific Blue anti-human CD45, dilution 1:100, #368540, BioLegend), 50 nM bis(1,3-dibutylbarbituric acid) trimethine oxonol (DiBAC_4_(3), #D8189, Sigma Aldrich, for MP), and 2.4 µM SNARF 5-(and-6)-carboxy-SNARF-1 (SNARF, #C1272, Invitrogen Thermo Fisher, Dreieich, Germany, for pH_i_). After 10 min of incubation in the dark at room temperature, the diluted blood was mixed with 950 µl Hanks’ Balanced Salt Solution (HBSS^++^, #14025-050, Gibco Thermo Fisher Scientific) adjusted to pH 7.3 including 50 nM DiBAC_4_(3) and transferred to a light-protected water bath at 37°C. After a resting period of 2 min, neutrophils were stimulated with either PBS^++^ (as buffer control), PAF or with the mixture of proinflammatory mediators described above. After exclusion of erythrocytes as CD45 negative cells, neutrophils were identified as described below. An increase in DiBAC_4_(3) indicates depolarization, and a decrease in PE/PerCP ratio in SNARF indicates alkalization, respectively (33–36).

For the analysis by flow cytometry, doublets were removed by plotting the forward scatter (FSC) area versus the height. Neutrophils and monocytes were identified on the basis of their forward and side scatter (SSC) area properties. The spillover between the fluorescence channels was corrected by a compensation matrix. For all antigens, appropriate isotype controls and single staining controls were performed (data not shown). For all experiments, a minimum of 3000 neutrophils and 500 monocytes were recorded using a BD FACSLyric (BD Biosciences). The gating strategy for the analysis of neutrophils and monocytes is illustrated in **Supplemental Figure 1**.

### 2.4 Flow cytometry analysis of platelets

For the brief analysis of platelet activation, 50 µl citrate-anticoagulated blood was diluted with 562.5 µl HBSS^++^ adjusted to pH 7.3. Hereafter, 10 µl of this diluted whole blood was added to 40 µl PBS^++^ adjusted to pH 7.3 including prior added stimuli (either PBS^++^ as Ctrl, PAF, or the mixture of proinflammatory mediators) and antibodies to CD61 (anti-CD61 PerCP mouse anti-human CD61, dilution 1:100, #336410, BioLegend) and CD62P (FITC anti-human CD62P (P-Selectin), dilution 1:25, #304904, BioLegend). Following incubation for 10 minutes in a light-protected water bath at 37°C, 950 µl HBSS^++^ were added to the sample followed by immediate flow cytometry analysis. Platelets were identified by the properties of FSC, SSC, and CD61 expression. The gating strategy for the analysis of thrombocytes is summarized in **Supplemental Figure 2**.

### 2.5 Determination of platelet-neutrophil complexes and platelet-monocyte complexes

PNCs were analyzed by light microscopy and flow cytometry as previously described (21, 33, 37). For analysis by light microscopy (Axio Imager M1, Carl Zeiss Microscopy GmbH, Jena, Germany), 250 µl citrate anticoagulated whole blood was diluted with 250 µl PBS^++^ adjusted to pH 7.3 and stimulated with either PBS^++^ as buffer control or 1 µM PAF. Blood smears were stained with the “Hemacolor Rapid staining of blood smear - staining set for microscopy” (Merck, Darmstadt, Germany). For each sample, a minimum of 50 neutrophils per specimen were analyzed by two independent and blinded individuals. Each neutrophil with at least one thrombocyte in direct juxtaposition was counted as a PNC. Representative PNCs identified by light microscopy are shown in **Supplemental Figure 8A**. The analysis of PNCs and PMCs by flow cytometry was conducted similarly to the staining protocol described in 2.3 using antibodies against CD61 (PerCP Mouse anti-human CD61, dilution 1:50, #336410, BioLegend) and CD42b (APC-Cyanine7 anti-human CD42b, dilution 1:400, #303920, BioLegend). An example of the resulting staining and corresponding gating strategy is given in **Supplemental Figure 8**.

### 2.6 Analysis of neutrophil chemotaxis

Polymorphonuclear granulocytes mainly consisting of neutrophils were isolated by Ficoll-Paque (GE Healthcare, Uppsala, Sweden) density gradient centrifugation and subsequent dextran sedimentation followed by hypotonic lysis of the remaining erythrocytes, as previously described (21, 33, 34, 36). Neutrophil chemotactic activity was analyzed using a Neuro Probe A96 chemotaxis chamber (Neuro Probe, Gaithersburg, USA). Isolated neutrophils at a concentration of 1 × 10^6^ cells/ml were suspended in HBSS^++^ adjusted to pH 7.3. Neutrophils were stained with the fluorescent dye BCECF (1.6 µl/ml, BCECF-AM, Abcam, Cambridge, United Kingdom) for 30 minutes at 37°C, subsequently centrifuged for 5 minutes (340 × g) and resuspended in HBSS^++^ + 0.1% BSA. A total of 33 µl chemoattractant PAF (final concentration 1 µM) or a mixture of PAF, fMLF, and TNF (final concentrations 1 µM PAF, 10 µM fMLF, and 2.3 µM TNF) was added to the wells of the lower plate. Subsequently, a silicone gasket and a framed filter with 3 µm pores (Neuro Probe) were placed on the lower wells. On top of the filter and the gasket, the upper plate was attached and the stained neutrophils were pipetted into the corresponding wells. During incubation for 30 minutes at 37°C, neutrophils migrated from the upper wells towards the lower wells containing the inflammatory stimuli, but became adherent to the filter, resulting in increased fluorescence. The fluorescence of the cells in the filter was determined at a wavelength of 485/538 nm using a Fluoroskan Ascent (Thermo Fisher Scientific) with Ascent Software Version 6.0.2.

### 2.7 Data analysis and statistics

The flow cytometry data including of neutrophils, monocytes, and platelets were further analyzed using the custom-written, python-based flow cytometry analytics software “BFlow” (BFlow Project, www.bflow.science, last accessed 28^th^ February 2023). All data is presented as medians with bars indicating the interquartile range, for example, median (25^th^ percentile|75^th^ percentile). **Figure 1B** was created with BioRender.com. Data analysis was performed with licensed versions of Microsoft Excel 2019 (Microsoft, Redmond, USA) and GraphPad Prism 9 (GraphPad Software Inc, San Diego, USA). For the statistical analysis comparing HV with patients with CF before the initiation of ETI treatment (T1), the data distribution was considered nonparametric and unpaired and analyzed using the Mann–Whitney U test. To compare the results of T1 and T2 (after 6 months of ETI treatment), the data distribution was considered nonparametric and analyzed by the Wilcoxon test for paired comparison (thereby automatically excluding patients who did not present at both T1 and T2). Categorical variables for T1 vs. T2 were analyzed using the Fisher exact test. A p-value < 0.05 was considered to be significant and marked with *, **, ***, or ****, indicating < 0.05, < 0.01, < 0.001, and <0.0001, respectively.

## 3 Results

### 3.1 Patient characteristics and clinical features

**Figure 1** and **Supplemental Tables 1**, **2** summarize the characteristics of the patients with CF before (T1) compared to the age- and sex- matched HV and after 6 months of ETI treatment (T2). The study group consisted initially of 13 patients with CF (T1) with a median age of 26 years and a male:female ratio of 6:7. Two patients were lost during follow-up, resulting in 11 patients at T2. In total, 11/13 patients (84.6%) were homozygous for p.Phe508del mutation, while 2/13 (15.4%) were heterozygous for p.Phe508del, with the second mutation determined to be rs1799022949 or rs121908751 (38). Prior to ETI treatment, 7/13 (53.9%) patients had already been treated with CFTR modulators (n = 6: Lumacaftor–Ivacaftor, n = 1: Ivacaftor).

### 3.2 ETI treatment alters markers of organ function, disease severity, and humoral inflammation

**Figure 2:**
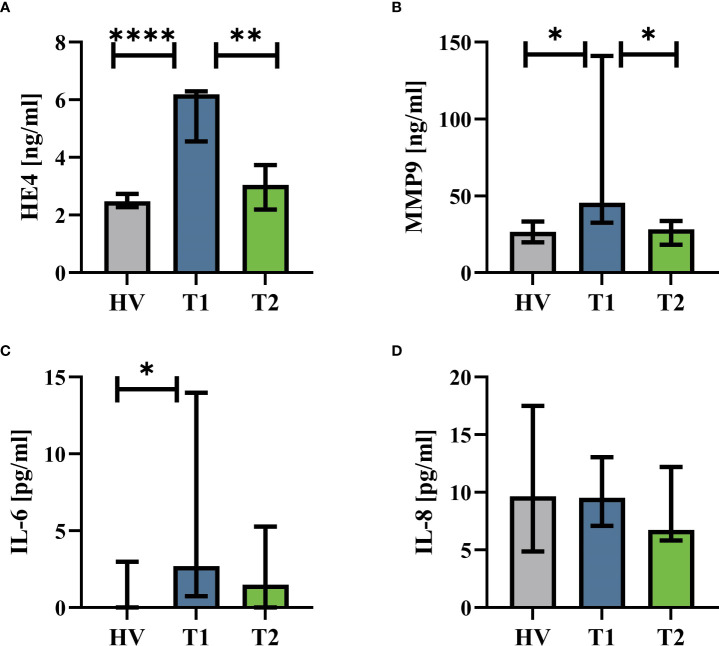
Humoral inflammatory markers in patients with CF before Elexacaftor–Tezacaftor–Ivacaftor (ETI) treatment (T1) compared to age- and sex- matched healthy volunteers (HV) and after 6 months of ETI treatment (T2). **(A)** Human epididymis protein 4 (HE4), **(B)** matrix metallopeptidase 9 (MMP9), **(C)** interleukin 6 (IL-6), and **(D)** interleukin 8 (IL-8). n = 11 – 13. Median with interquartile range. *, **, **** denote p < 0.05, 0.01, and 0.0001, respectively.

The patients with CF had normal AST (T1: 23 U/l (19|16) vs. T2: 26 U/L (23|32), p = 0.82), ALT (T1: 23 U/L (15|27) vs. T2: 31 U/L (23|43), p = 0.09), and creatinine values (T1: 63 U/L (54|74) vs. T2: 63 U/L (49|78), p = 0.75). **Supplemental Table 1** summarizes the complete blood counts at T1 and T2. ETI treatment increased the BMI of the patients, reduced the sweat chloride concentration, and improved lung function (**Figure 1A**). Furthermore, HE4 was significantly increased in T1 (despite normal renal function, data not shown) compared to HV but also significantly reduced in T2 (**Figure 2**). As a first step to monitor possible changes in inflammation, humoral markers of inflammation were analyzed. Here, patients with CF at T1 had slightly but significantly elevated MMP9 and IL-6 levels compared to HV (**Figure 2**). At T2, MMP9 was significantly and IL-6 was trendwise reduced. IL-8 (**Figure 2**) did not show significant changes.

### 3.3 Neutrophils and monocytes in patients with CF remain unaffected regardless of ETI treatment

**Figure 3:**
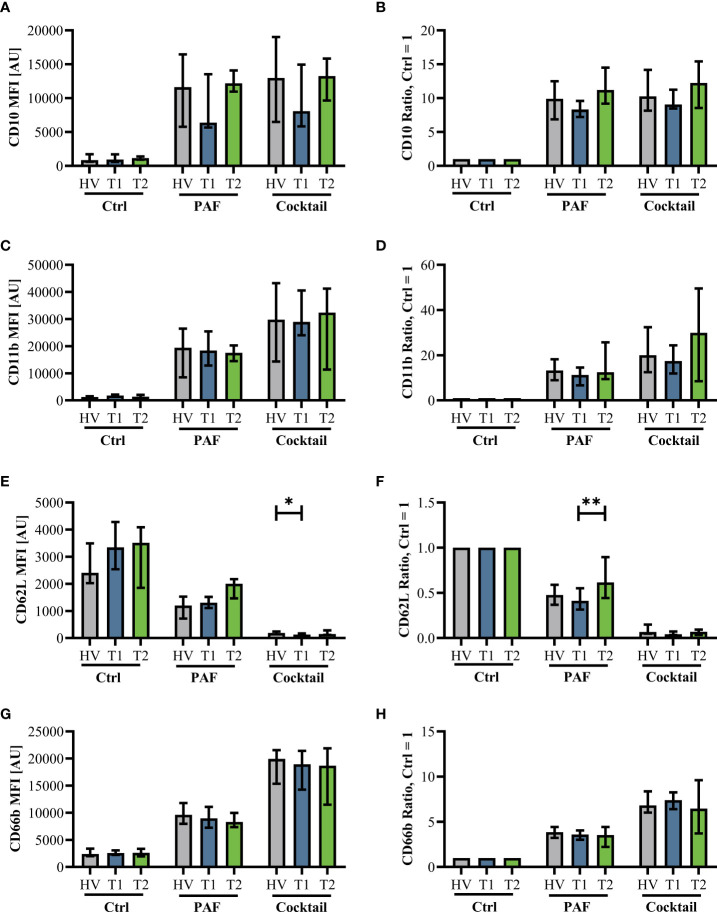
Neutrophil activation markers in patients with CF before Elexacaftor–Tezacaftor–Ivacaftor (ETI) treatment (T1) compared to age- and sex- matched healthy volunteers (HV) and after 6 months of ETI treatment (T2). The left panel shows median fluorescence intensity (MFI) values. The right panel shows normalization of the neutrophils stimulated with 1 µM PAF or a mixture of proinflammatory mediators (Cocktail: 1 µM PAF, 10 µM fMLF, 2.3 µM TNF) normalized to the respective cells exposed to a buffer control (Ctrl = 1). **(A, B)**: CD10, **(C, D)**: CD11b, **(E, F)**: CD62L, and **(G, H)**: CD66b. Data of corresponding experiments with further stimuli are given in **Supplemental Figure 3**. n = 11 – 13, median with interquartile range. * and ** denote p < 0.05 and 0.01, respectively.

**Figure 4:**
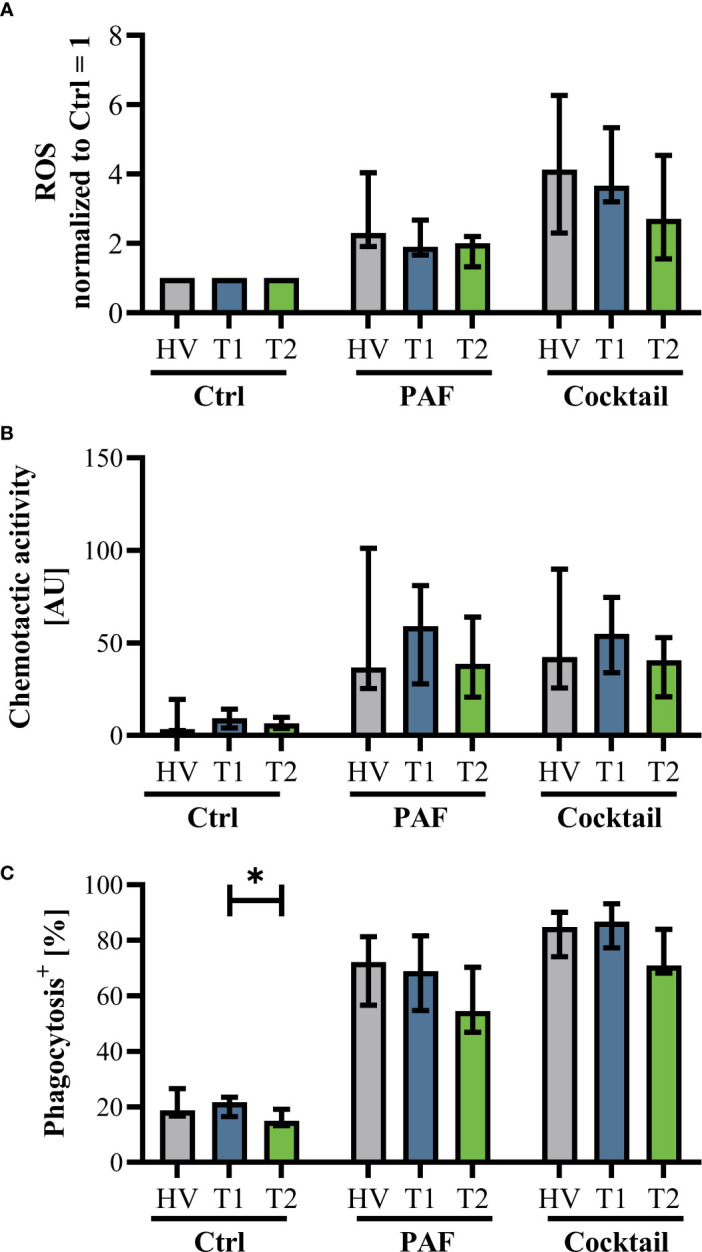
Markers of neutrophil function in patients with CF before Elexacaftor–Tezacaftor–Ivacaftor (ETI) treatment (T1) in comparison to age- and sex- matched healthy volunteers (HV) and after 6 months of ETI treatment (T2). **(A)** Generation of radical oxygen species (ROS) after stimulation with PAF or a mixture of proinflammatory mediators (Cocktail: 1 µM PAF, 10 µM fMLF, 2.3 µM TNF) normalized to the respective samples exposed to buffer control, **(B)** chemotactic activity of neutrophils, and **(C)** phagocytic activity. Data of corresponding experiments with further stimuli are given in **Supplemental Figure 6**. n = 11 – 13, median with interquartile range. * denotes p < 0.05.

Innate immunity was monitored by analyzing neutrophil cell physiology, phenotype, and function as well as monocyte phenotype. Neutrophil phenotype and cell physiology was largely similar comparing patients with CF at T1 and HV (**Figure 3**; **Supplemental Figure 3**). In accordance, ETI treatment did not result in corresponding alterations in the neutrophil phenotype at T2 (**Figure 3**; **Supplemental Figure 3**). A similar pattern was observed in monocytes (**Supplemental Figures 4**, **5**). Of note, the cellular response to additional stimulation in vitro was slightly increased at T2 for neutrophil CD62L expression as well as for monocyte CD10, CD11b, and CD62L expression. Neutrophil function and cell physiology was also comparable when analyzing HV and T1 with respect to ROS generation, chemotactic activity, and phagocytosis (**Figure 4**; **Supplemental Figure 6**). At T2, baseline chemotactic activity and ROS generation remained stable. Anyhow, upon additional stimulation, ROS generation and chemotactic activity of neutrophil were unchanged. However, there was a small but significant decrease in phagocytic activity. Likewise, cellular physiology as indicated by changes in MP and pH_i_ upon stimulation were similar comparing HV and patients with CF and remained unaffected by ETI treatment (**Supplemental Figure 7**).

The activity of platelets as reported by CD62P under resting conditions or after stimulation with PAF was similar when analyzing HV, T1, and T2 (**Supplemental Figure 8**). Likewise, the formation of PNCs and PMCs (activated platelets adhering to neutrophils or monocytes) was comparable when analyzing HV, T1, and T2 (**Supplemental Figure 8**). Of note, the response to LPS regarding the formation of PNCs and PMCs was slightly increased at T1 but not T2 in comparison to HV, however, with a small effect size.

## 4 Discussion

This study investigated the function and the phenotype of cellular innate immunity with a focus on neutrophils in patients with CF before and after with ETI treatment in comparison to healthy volunteers. In accordance with data from the original phase III clinical trial (4) and postadmission studies (39), ETI treatment improved lung function, decreased sweat chloride, and increased the BMI, indicating a relevant clinical effect within the study population. In accordance, HE4 as an inflammatory biomarker (40, 41) decreased during the study period, which was also reflected by a slight, yet significant decrease in MMP9.

In accordance with a recent study (20), neutrophils from patients with stable CF had a similar phenotype in comparison to those from HVs. Moreover, the present study showed that neutrophils from patients with CF were able to change their phenotype to additional stimulation in vitro similarly as neutrophils from HVs. This is of interest because neutrophils previously exposed to lipopolysaccharide displayed a diminished response to additional stimulation in vitro (21, 22).

In contrast to the well-known causality between CFTR dysfunction and defective epithelial chloride transport, it remains a matter of debate whether CFTR directly affects cellular innate immune function. The CFTR protein was detected in the phagolysosome of human neutrophils (42). Interestingly, neutrophils derived from patients with CF showed impaired phagosomal chlorination and bacterial killing, indicating an intrinsic phagocyte defect in neutrophils from patients with CF. CFTR mRNA and protein levels in neutrophils and other phagocyting cells were reported to be very low (43). However, a recent study found significantly reduced CFTR protein expression levels in CF MDMs, which were restored by ETI treatment (44). Therefore, it remains an ongoing debate as to whether the reported antimicrobial impairment of neutrophils from patients with CF is intrinsic or secondary to abnormalities in the microenvironment of the apical surface liquid of the airways of such patients or the result of continuous inflammation and infection (5, 12). The present study did not find differences in phagocytosis measured as uptake of microspheres between neutrophils from patients with CF and HV. However, the present study did not determine phagosomal chlorination or bacterial killing with the used method.

The role of CFTR modulators in innate immune cell function has been previously studied. IVA treatment improved bacterial killing in neutrophils and monocyte-derived macrophages (45, 46). Moreover, IVA treatment resulted in an altered activation profile with a decrease in activated CD11b in peripheral blood mononuclear cells from patients with the G551D mutation but not in cells from patients with p.Phe508del (47). The corrector Lumacaftor also improved phagocytosis and bacterial killing (48). However, its combination with IVA failed to restore phagocytic function, but did reduce the secretion of proinflammatory cytokines (48, 49). Similar effects have been reported for the combination of TEZ and IVA (50). Regarding the heterogeneous results, it is unclear whether the reported effects are drug specific, mutation specific, or depend on the degree of CFTR restoration, as reviewed in (30). The impact of the latest CFTR modulator combination ETI on cellular innate immunity is largely unknown. In monocytes, reduced inflammasome activity (51) and increased phagocytic activity (29) were reported after ETI treatment. Currently, the present work is, to our knowledge, the first study that focuses on neutrophil phenotype and function in resting or stimulated cells after ETI treatment in patients with CF.

The present article has several strengths and limitations. Despite that there were some significant changes in cellular innate immunity, the authors interpreted the reported changes as likely to be without clinical consequences. The findings also indicated that ETI treatment had no negative effect on cellular innate immunity. Moreover, while the study population mimicked the typical characteristics of CF in general and the ETI treatment in particular, patients with CF were investigated during stable periods of disease without exacerbation in a monocentric prospective study. Therefore, we may have missed changes in neutrophil and/or monocyte phenotype and/or function, which potentially only become apparent during acute exacerbation (20). However, distinguishing these distinct alterations from the general characteristics of acute inflammation (e.g., during sepsis in patients without CF) was beyond the scope of the present study. To partially account for this limitation, neutrophils were additionally stimulated in vitro with clinically relevant proinflammatory mediators, revealing the IVA-mediated changes in the neutrophil response. Innate immunity was thoroughly analyzed by monitoring neutrophil phenotype and function as well as by briefly monitoring markers of characterizing activation and the formation of platelet-neutrophil complexes as an indirect surrogate of platelet activation. However, the focus on innate immunity only represents certain aspects of immunity.

## 5 Conclusion

Patients with CF are affected by multiple severe organ function alterations, which in this study did not affect circulating innate immunity and only marginally humoral markers of inflammation. While this study confirmed previous beneficial effects of ETI on clinical data and markers of humoral inflammation, no major effects on innate immunity were detected, besides some alterations after additional stimulation in vitro with inflammatory mediators. Nevertheless, ETI treatment did not impair cellular innate immunity. The present study population consisted of patients with currently well-controlled CF. Further studies are needed to evaluate potential benefits of ETI treatment during acute exacerbation in patients with CF. In summary, in patients with CF without acute exacerbation, circulating innate immunity did not exhibit any alterations.

## Data availability statement

The original contributions presented in this study are included in the article/**Supplementary Material**. Further inquiries can be directed to the corresponding author.

## Ethics statement

The studies involving human participants were reviewed and approved by Local Independent Ethics Committee of the University of Ulm. The patients/participants provided their written informed consent to participate in this study.

## Author contributions

Conceptualization: HS, MH-L, and DM. Data curation: HS, LH, LW, and DM. Formal analysis: HS, LH, LW, and DM. Funding acquisition: MH-L. and DM. Investigation: HS, LH, LW, CK, AM, LS, FM, AS, CB, PM, and DM. Methodology: HS, LH, LW, CK, and DM. Project administration: MH-L and DM. Resources: MH-L and DM. Visualization: LH and DM. Writing – original draft: HS, LH, and DM. Writing - review & editing: all authors. All authors contributed to the article and approved the submitted version.

## Supplementary material

Supplementary Table 1 Complete blood count in patients with CF before Elexacaftor–Tezacaftor–Ivacaftor (ETI) treatment (T1) and after 6 months of ETI treatment (T2), n = 11 – 13, * = p < 0.05.

Supplementary Table 2 Results of the microbial monitoring in patients with CF before Elexacaftor–Tezacaftor–Ivacaftor (ETI) treatment (T1) and after 6 months of ETI treatment (T2), n = 11 – 13, * = p < 0.05.

Supplementary Figure 1 Representative gating strategy for neutrophils and monocytes. **(A)** Identification of single cells by analyzing forward scatter (FSC) area versus the height. **(B)** neutrophils and monocytes were identified based on their forward and side scatter (SSC) area properties. **(C)** Example of changes in neutrophil, CD11b expression in unstained cells (native, gray), after exposure to the buffer control (dark yellow, Ctrl), or a mixture of proinflammatory mediators consisting of 1 µM PAF, 10 µM fMLF, and 2.3 µM TNF (Cocktail, red). **(D)** Exemplary analysis of the percentage of neutrophils with phagocytic activity as measured by uptake of fluorescent beads.

Supplementary Figure 2 Representative gating strategy for thrombocytes. **(A)** Identification of small particles including thrombocytes. **(B)** Identification of single particles by analyzing forward scatter (FSC) area versus the height. **(C)** Identification of platelets as CD61 positive entities. **(D)** Exemplary analysis of the percentage of platelets positive for CD62P in samples stained with an antibody against CD61 and the isotype control for CD62P (Isotype, purple), after exposure to the buffer control (dark yellow, Ctrl), or a mixture of proinflammatory mediators consisting of 1 µM PAF, 10 µM fMLF, and 2.3 µM TNF (Cocktail, red).

Supplementary Figure 3 Neutrophil activation markers in patients with CF before Elexacaftor–Tezacaftor–Ivacaftor (ETI) treatment (T1) compared to age- and sex- matched healthy volunteers (HV) and after 6 months of ETI treatment (T2). The left panel shows median fluorescence intensity (MFI) values. The right panel shows normalization of the neutrophils stimulated with 1 µM fMLF, 100 ng/ml LPS from Escherichia coli (LPS EC), or 1 µg/mL LPS from Pseudomonas aeruginosa (LPS PsA) normalized to the respective cells exposed to a buffer control (Ctrl = 1). **(A, B)** CD10, **(C, D)** CD11b, **(E, F)** CD62L, and **(G, H)**: CD66b. n = 11 – 13, median with interquartile range. * denotes p < 0.05.

Supplementary Figure 4 Monocyte activation markers in patients with CF before Elexacaftor–Tezacaftor–Ivacaftor (ETI) treatment (T1) compared to age- and sex- matched healthy volunteers (HV) and after 6 months of ETI treatment (T2). The left panel shows median fluorescence intensity (MFI) values. The right panel displays normalization of the neutrophils stimulated with 1 µM PAF or a mixture of proinflammatory mediators (Cocktail: 1 µM PAF, 10 µM fMLF, 2.3 µM TNF) normalized to the respective cells exposed to a buffer control (Ctrl = 1). **(A, B)** CD10, **(C, D)** CD11b, and **(E, F)** CD62L. n = 11 – 13, median with interquartile range. *, **, ***, ****, denote p < 0.05, 0.01, 0.001, and 0.0001, respectively.

Supplementary Figure 5 Monocyte activation markers in patients with CF before Elexacaftor–Tezacaftor–Ivacaftor (ETI) treatment (T1) compared to age- and sex- matched healthy volunteers (HV) and after 6 months of ETI treatment (T2). The left panel shows median fluorescence intensity (MFI) values. The right panel shows normalization of the neutrophils stimulated with 1 µM fMLF, 100 ng/ml LPS from Escherichia coli (LPS EC), or 1 µg/mL LPS from Pseudomonas aeruginosa (LPS PsA) normalized to the respective cells exposed to a buffer control (Ctrl = 1). **(A, B)**: CD10, **(C, D)**: CD11b, and **(E, F)**: CD62L. n = 11 – 13, median with interquartile range. *, **, *** denote p < 0.05, 0.01 and 0.001, respectively.

Supplementary Figure 6 Markers of neutrophil function in patients with CF before Elexacaftor–Tezacaftor–Ivacaftor (ETI) treatment (T1) in comparison to age- and sex- matched healthy volunteers (HV) and after 6 months of ETI treatment (T2). **(A)** Generation of radical oxygen species (ROS) after stimulation with 1 µM fMLF, 100 ng/ml LPS from Escherichia coli (LPS EC), or 1 µg/mL LPS from Pseudomonas aeruginosa (LPS PsA) normalized to the respective samples exposed to buffer control, **(B)** chemotactic activity of neutrophils, and **(C)** phagocytic activity. n = 11 – 13, median with interquartile range. * denotes p < 0.05.

Supplementary Figure 7 Markers of neutrophil cell physiology in patients with CF before Elexacaftor–Tezacaftor–Ivacaftor (ETI) treatment (T1) in comparison to age- and sex- matched healthy volunteers (HV) and after 6 months of ETI treatment (T2). **(A)** Intracellular pH (decrease in PE/PerCP ratio = alkalization) and **(B)** membrane potential (increase in DiBAC fluorescence = depolarization) after stimulation with PAF or a mixture of proinflammatory mediators (Cocktail: 1 µM PAF, 10 µM fMLF, 2.3 µM TNF). n = 11 – 13, median with interquartile range.

Supplementary Figure 8 Formation of platelet-neutrophil complexes (PNCs) and platelet-monocyte complexes (PMCs) in patients with CF before Elexacaftor–Tezacaftor–Ivacaftor (ETI) treatment (T1) in comparison to age- and sex- matched healthy volunteers (HV) and after 6 months of ETI treatment (T2). The formation of PNCs and PMCs was analyzed in diluted whole blood after 15 minutes of 13 timulation with PBS as buffer control or stimulated with 1 µM PAF. **(A)** Representative neutrophil and PNC as detected by light microscopy. **(B)** Evaluation of PNCs by light microscopy. **(C)** Representative histogram of the CD61 signal in neutrophils showing the two populations (neutrophils with or without platelets) in dependence of previous stimulation with PAF (orange), LPS EC (pink), LPS PsA (purple), or buffer control (Ctrl, gray). Analysis of PNC formation by flow cytometry using **(D)** CD61 (as shown in **(C)**) on neutrophils or **(E)** CD42b on neutrophils as markers of PNCs. **(F)** Analysis of CD62P-expression on thrombocytes as a marker of thrombocyte activation. **(G)** Representative histogram of the CD61 signal in monocytes showing the two populations (monocytes with or without platelets) in dependence of previous stimulation. **(H)** Analysis of PMC formation by flow cytometry as shown in **(G)** using CD61 on monocytes PAF = platelet-activating factor, LPS EC = lipopolysaccharide from Escherichia coli, LPS PsA = lipopolysaccharide from Pseudomonas aeruginosa. n = 11 – 13, median with interquartile range. * denotes p < 0.05.
