# Supplementary material for: Multimodal analysis of granulocytes, monocytes, and platelets in patients with cystic fibrosis before and after Elexacaftor–Tezacaftor–Ivacaftor treatment
Source: Front Immunol. 2023 Jun 29;14:1180282. doi: 10.3389/fimmu.2023.1180282 (PMC10347380; doi:10.3389/fimmu.2023.1180282)
Supplement: Supplementary Table 1 — Complete blood count in patients with CF before Elexacaftor–Tezacaftor–Ivacaftor (ETI) treatment (T1) and after 6 months of ETI treatment (T2), n = 11 – 13, * = p < 0.05. [file DataSheet_1.pdf]

Supplementary Material

Multimodal analysis of granulocytes, monocytes, and platelets in patients with cystic fibrosis before and after Elexacaftor–Tezacaftor–Ivacaftor treatment

Hanna Schmidt[1,#], Larissa Melina Höpfer[2,#], Lisa Wohlgemuth[2], Christiane Leonie Knapp[2], Adam Omar Khalaf Mohamed[2], Laura Stukan[2], Frederik Münnich[2], Dominik Hüsken[2], Alexander Sebastian Koller[2], Alexander Elias Paul Stratmann[2], Paul Müller[2], Christian Karl Braun[1,3,4], Dorit Fabricius[1], Sebastian Felix Nepomuk Bode[1], Markus Huber-Lang[2], David Alexander Christian Messerer[2,5,*]

[1]Department of Pediatric and Adolescent Medicine, University Hospital Ulm, Ulm, Germany

[2]Institute of Clinical and Experimental Trauma Immunology, University Hospital Ulm, Ulm, Germany

[3]Institute of Transfusion Medicine, Ulm University, Ulm, Germany

[4]Institute of Clinical Transfusion Medicine and Immunogenetics Ulm, German Red Cross Blood Transfusion Service and University Hospital Ulm, Ulm, Germany

[5]Department of Transfusion Medicine and Hemostaseology, Friedrich-Alexander University Erlangen-Nuremberg, University Hospital Erlangen, Erlangen, Germany

[#] Hanna Schmidt and Larissa Melina Höpfer share the position as first author.

Parameter	Unit	T1	T2	T1 vs. T2
**Hemoglobin**	g/dl	13.9 (13.1|15.8)	14.4 (14.1|15.1)	0.81
**Leucocytes**	$\times 10^9$/l	6.3 (5.7|10.6)	6.6 (5.6|7.3)	*
**Thrombocytes**	$\times 10^9$/l	310 (259|374)	280 (257|292.5)	*
**Lymphocytes**	$\times 10^9$/l	1.7 (1.5|2.1)	1.8 (1.4|1.9)	0.25
**Neutrophil granulocytes**	$\times 10^9$/l	4.2 (3.6|7.9)	4.2 (3.0|4.6)	*
**Basophil granulocytes**	$\times 10^9$/l	0.04 (0.03|0.07)	0.04 (0.03|0.05)	0.13
**Eosinophil granulocytes**	$\times 10^9$/l	0.16 (0.11|0.21)	0.2 (0.11|0.29)	0.81
**Monocytes**	$\times 10^9$/l	0.5 (0.4|0.7)	0.5 (0.5|0.6)	0.41

Supplemental Table 1: Complete blood count in patients with CF before Elexacaftor–Tezacaftor–Ivacaftor (ETI) treatment (T1) and after 6 months of ETI treatment (T2), n = 11 – 13, * = $p < 0.05$.

	T1	T2	T1 vs. T2
**Achromobacter xylosidans**	1 (8%)	1 (9%)	>0.99
**Apsergillus fumigatus**	6 (46%)	0 (0%)	*
**Bordetella bronchiseptica**	0 (0%)	1 (9%)	0.46
**Burkholderia multivorans**	2 (15%)	1 (9%)	>0.99
**Candida species**	7 (54%)	7 (64%)	0.70
**Delftia acidovorans**	1 (8%)	0 (0%)	>0.99
**Enterobacter cloacae**	0 (0%)	1 (9%)	0.46
**Escherichia coli**	1 (8%)	0 (0%)	>0.99
**Haemophilus influenzae**	6 (46%)	1 (9%)	0.08
**Penicillinium species**	0 (0%)	1 (9%)	0.46
**Peroneutypa scoparia**	1 (8%)	0 (0%)	>0.99
**Pseudomonas aeruginosa**	5 (38%)	4 (36%)	>0.99
**Pseudomonas species (non-aeruginosa)**	0 (0%)	1 (9%)	0.46
**Staphylococcus aureus**	8 (62%)	7 (64%)	>0.99
**Stenotrophomonas maltophila**	1 (8%)	1 (9%)	>0.99
**Streptococcus agalactiae**	1 (8%)	0 (0%)	>0.99

Supplemental Table 2: Results of the microbial monitoring in patients with CF before Elexacaftor–Tezacaftor–Ivacaftor (ETI) treatment (T1) and after 6 months of ETI treatment (T2), n = 11 – 13, * = $p < 0.05$.

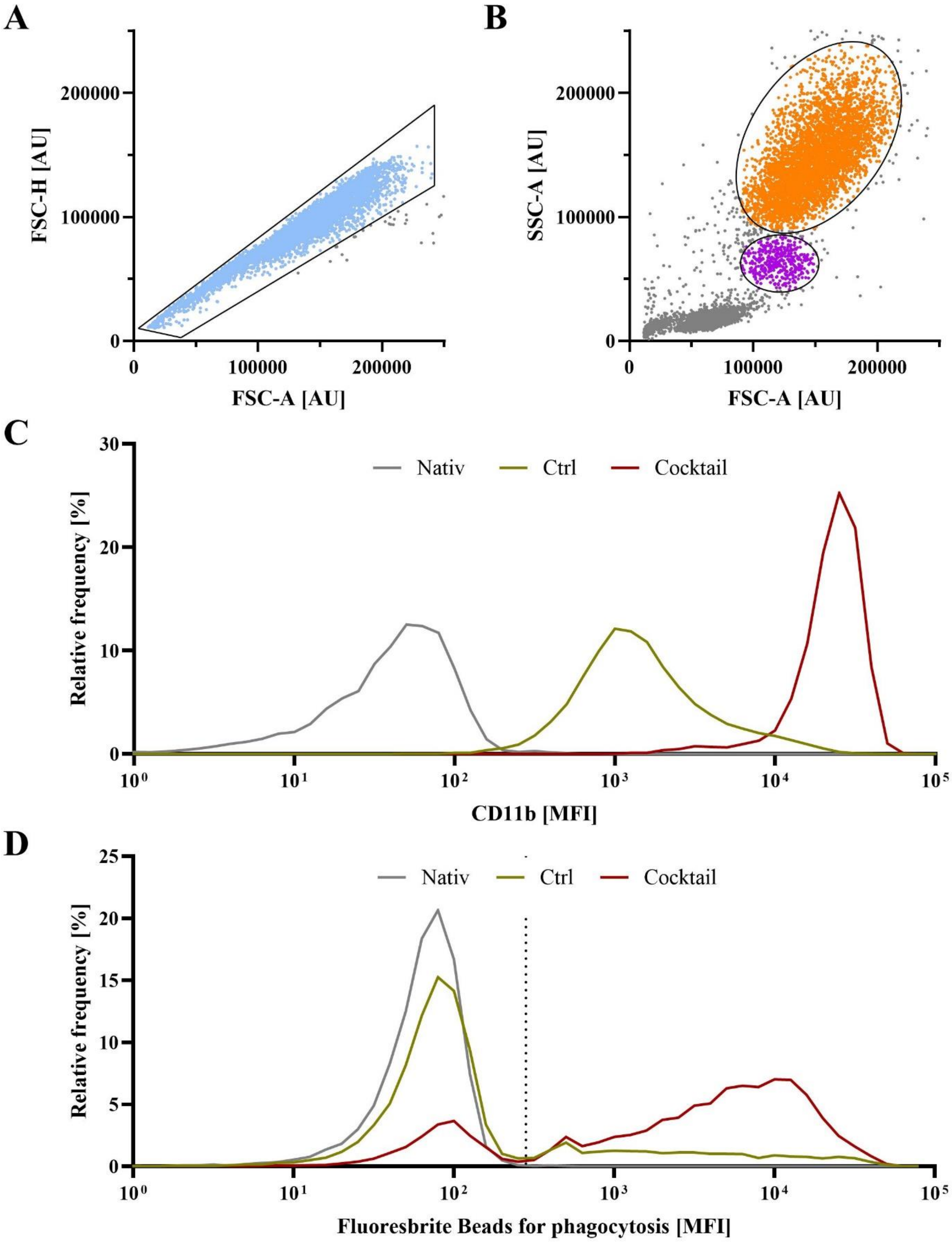

Supplemental Figure 1: Representative gating strategy for neutrophils and monocytes. **(A)** Identification of single cells by analyzing forward scatter (FSC) area versus the height. **(B)** neutrophils and monocytes were identified based on their forward and side scatter (SSC) area properties. **(C)** Example of changes in neutrophil, CD11b expression in unstained cells (native, gray), after exposure to the buffer control (dark yellow, Ctrl), or a mixture of proinflammatory mediators consisting of 1 µM PAF, 10 µM fMLF, and 2.3 µM TNF (Cocktail, red). **(D)** Exemplary analysis of the percentage of neutrophils with phagocytic activity as measured by uptake of fluorescent beads.

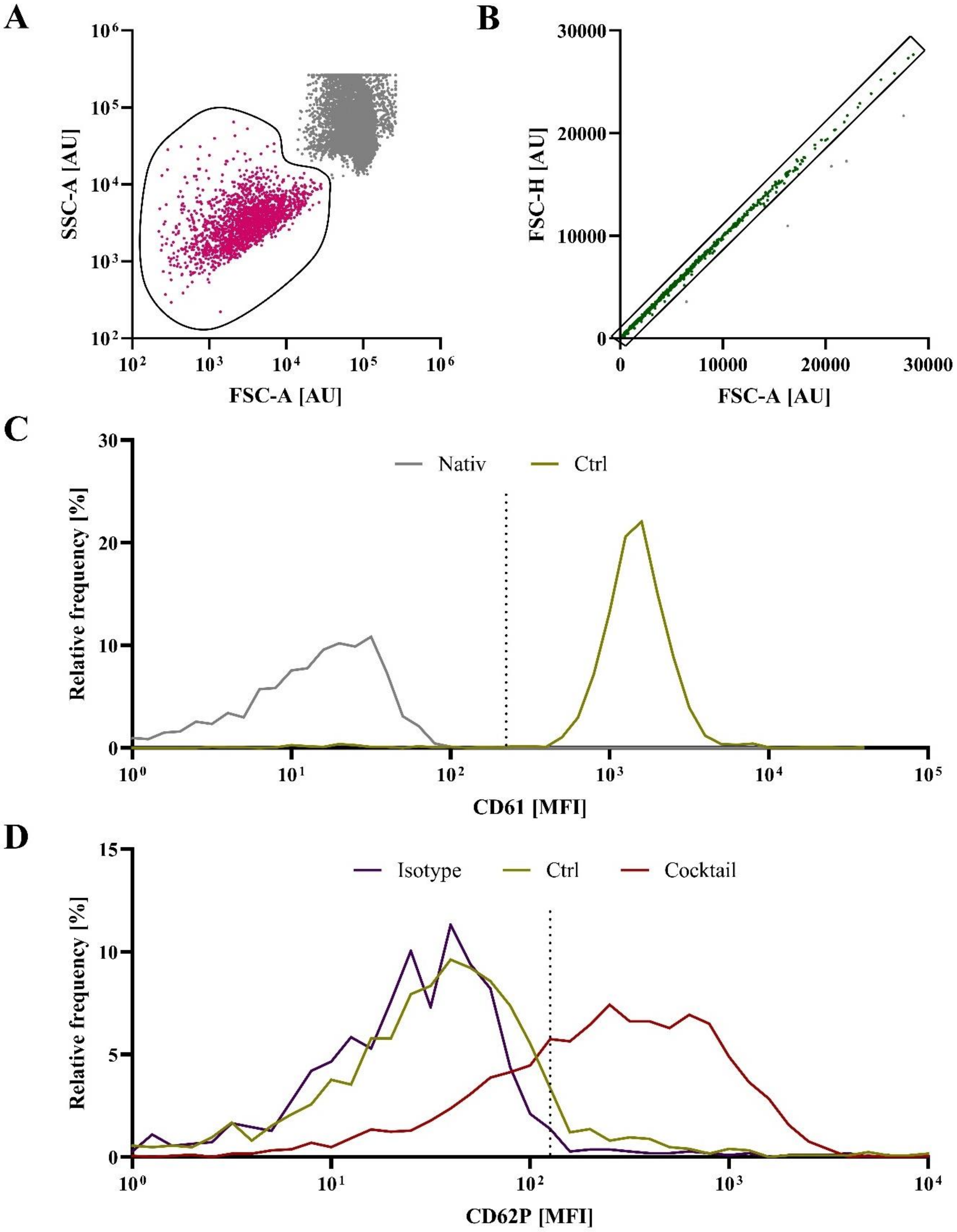

Supplemental Figure 2: Representative gating strategy for thrombocytes. **(A)** Identification of small particles including thrombocytes. **(B)** Identification of single particles by analyzing forward scatter (FSC) area versus the height. **(C)** Identification of platelets as CD61 positive entities. **(D)** Exemplary analysis of the percentage of platelets positive for CD62P in samples stained with an antibody against CD61 and the isotype control for CD62P (Isotype, purple), after exposure to the buffer control (dark yellow, Ctrl), or a mixture of proinflammatory mediators consisting of 1 µM PAF, 10 µM fMLF, and 2.3 µM TNF (Cocktail, red).

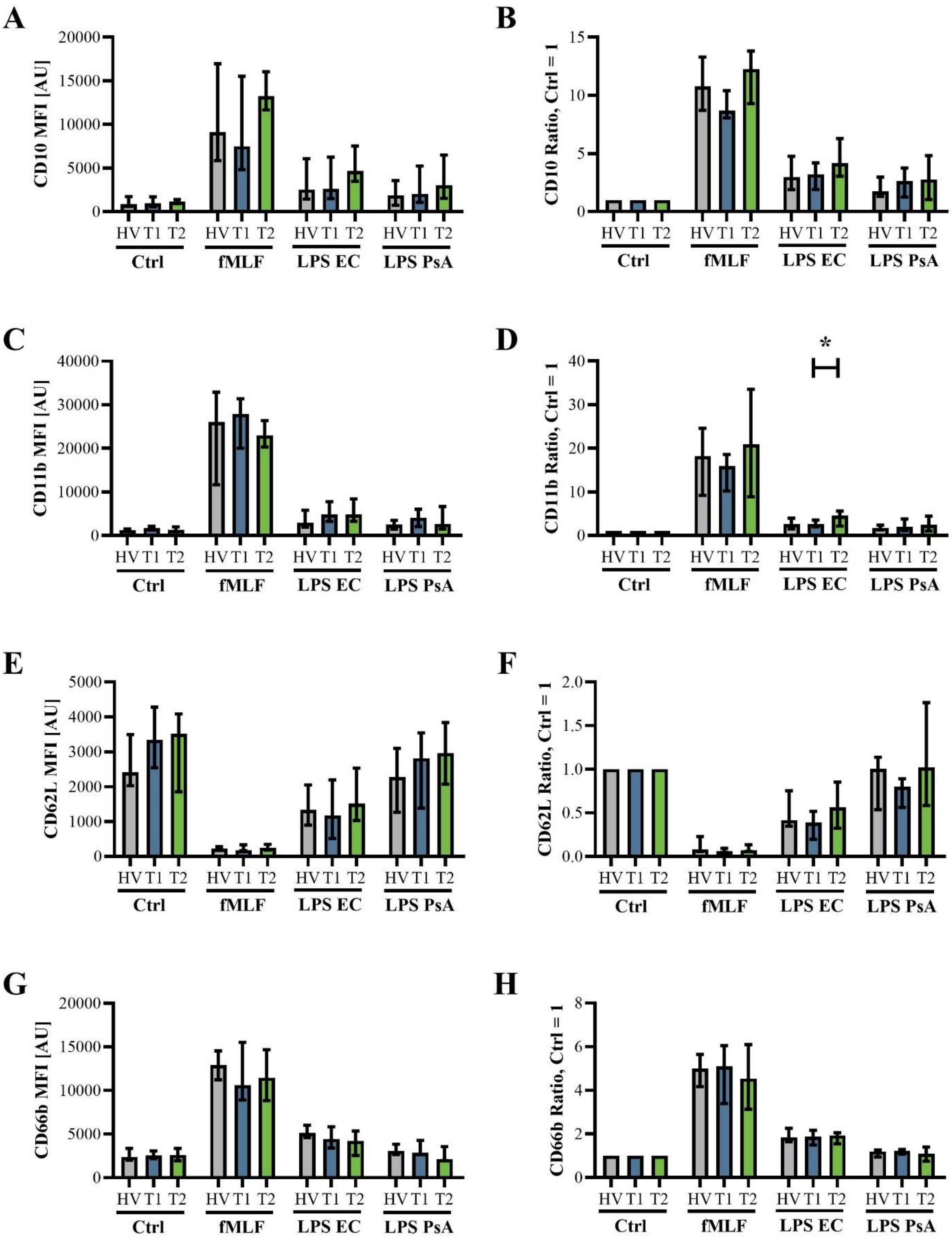

Supplemental Figure 3: Neutrophil activation markers in patients with CF before Elexacaftor–Tezacaftor–Ivacaftor (ETI) treatment (T1) compared to age- and sex- matched healthy volunteers (HV)

and after 6 months of ETI treatment (T2). The left panel shows median fluorescence intensity (MFI) values. The right panel shows normalization of the neutrophils stimulated with 1 µM fMLF, 100 ng/ml LPS from Escherichia coli (LPS EC), or 1 µg/mL LPS from Pseudomonas aeruginosa (LPS PsA) normalized to the respective cells exposed to a buffer control (Ctrl = 1). **(A, B)**: CD10, **(C, D)**: CD11b, **(E, F)**: CD62L, and **(G, H)**: CD66b. n = 11 – 13, median with interquartile range. * denotes $p < 0.05$.

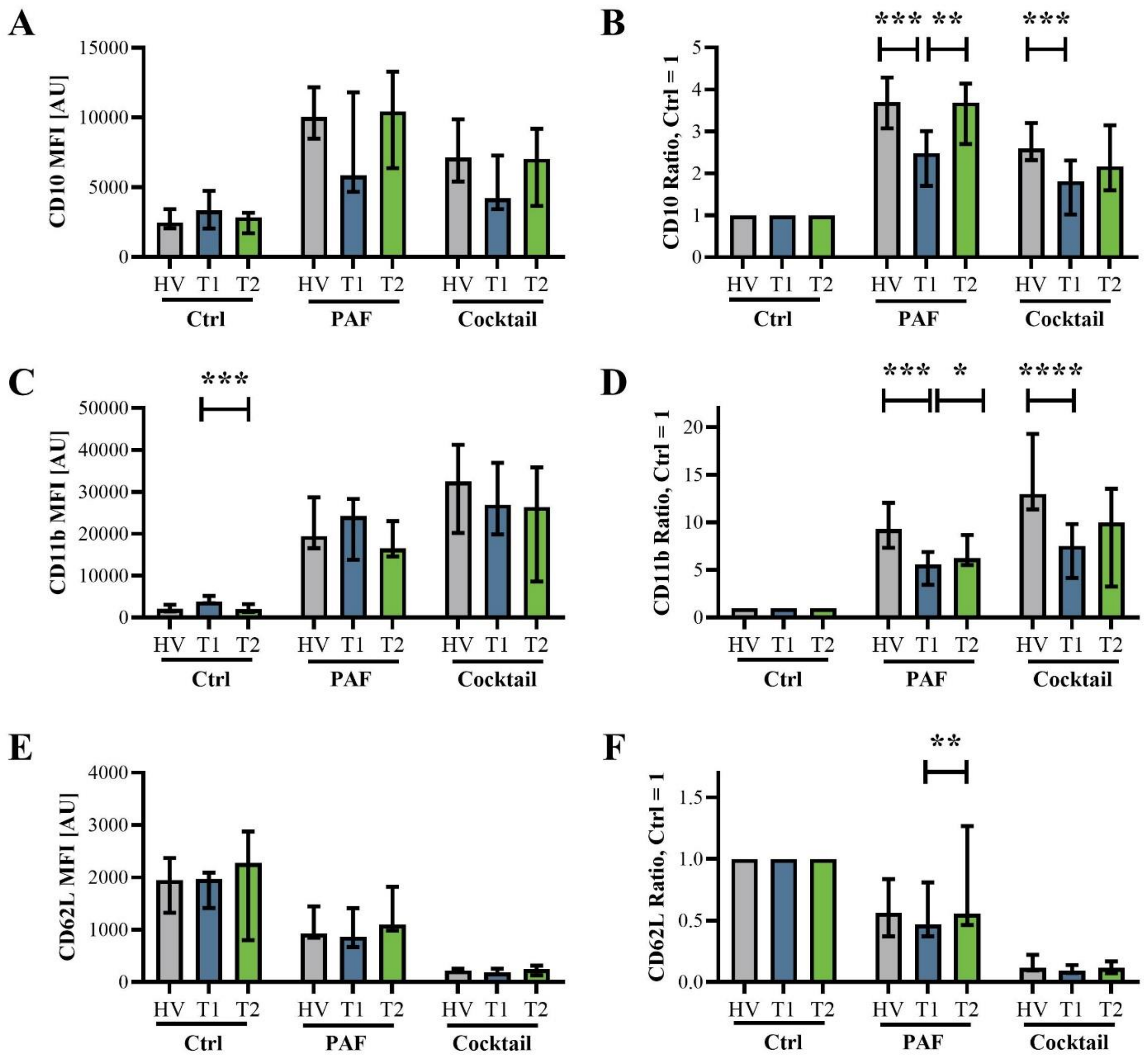

Supplemental Figure 4: Monocyte activation markers in patients with CF before Elexacaftor–Tezacaftor–Ivacaftor (ETI) treatment (T1) compared to age- and sex- matched healthy volunteers (HV) and after 6 months of ETI treatment (T2). The left panel shows median fluorescence intensity (MFI) values. The right panel displays normalization of the neutrophils stimulated with 1 µM PAF or a mixture of proinflammatory mediators (Cocktail: 1 µM PAF, 10 µM fMLF, 2.3 µM TNF) normalized to the respective cells exposed to a buffer control (Ctrl = 1). **(A, B)**: CD10, **(C, D)**: CD11b, and **(E, F)**: CD62L. n = 11 – 13, median with interquartile range. *, **, ***, ****, denote $p < 0.05$, 0.01, 0.001, and 0.0001, respectively.

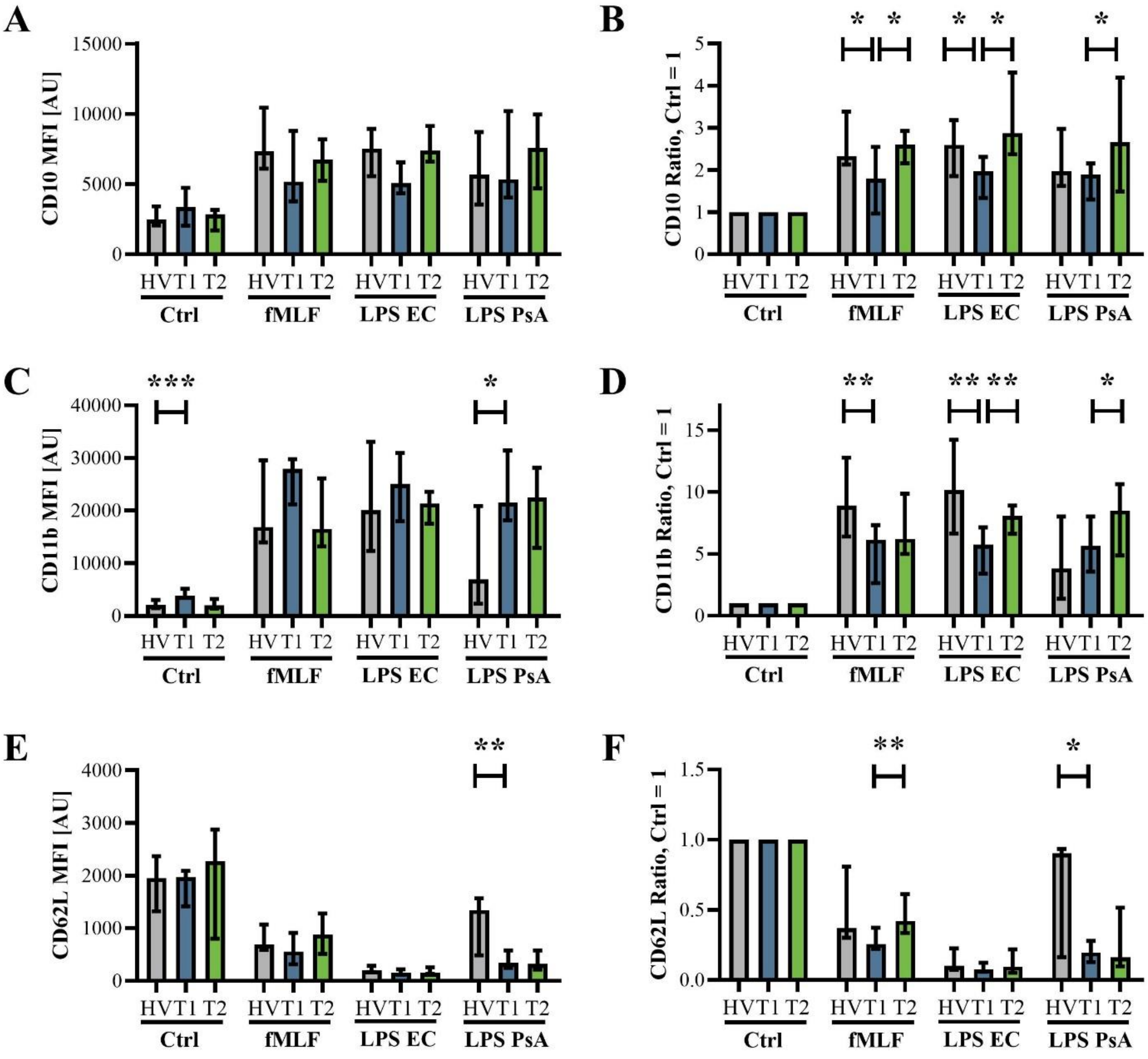

Supplemental Figure 5: Monocyte activation markers in patients with CF before Elexacaftor–Tezacaftor–Ivacaftor (ETI) treatment (T1) compared to age- and sex- matched healthy volunteers (HV) and after 6 months of ETI treatment (T2). The left panel shows median fluorescence intensity (MFI) values. The right panel shows normalization of the neutrophils stimulated with 1 µM fMLF, 100 ng/ml LPS from Escherichia coli (LPS EC), or 1 µg/mL LPS from Pseudomonas aeruginosa (LPS PsA) normalized to the respective cells exposed to a buffer control (Ctrl = 1). **(A, B)**: CD10, **(C, D)**: CD11b, and **(E, F)**: CD62L. n = 11 – 13, median with interquartile range. *, **, *** denote $p < 0.05$, 0.01 and 0.001, respectively.

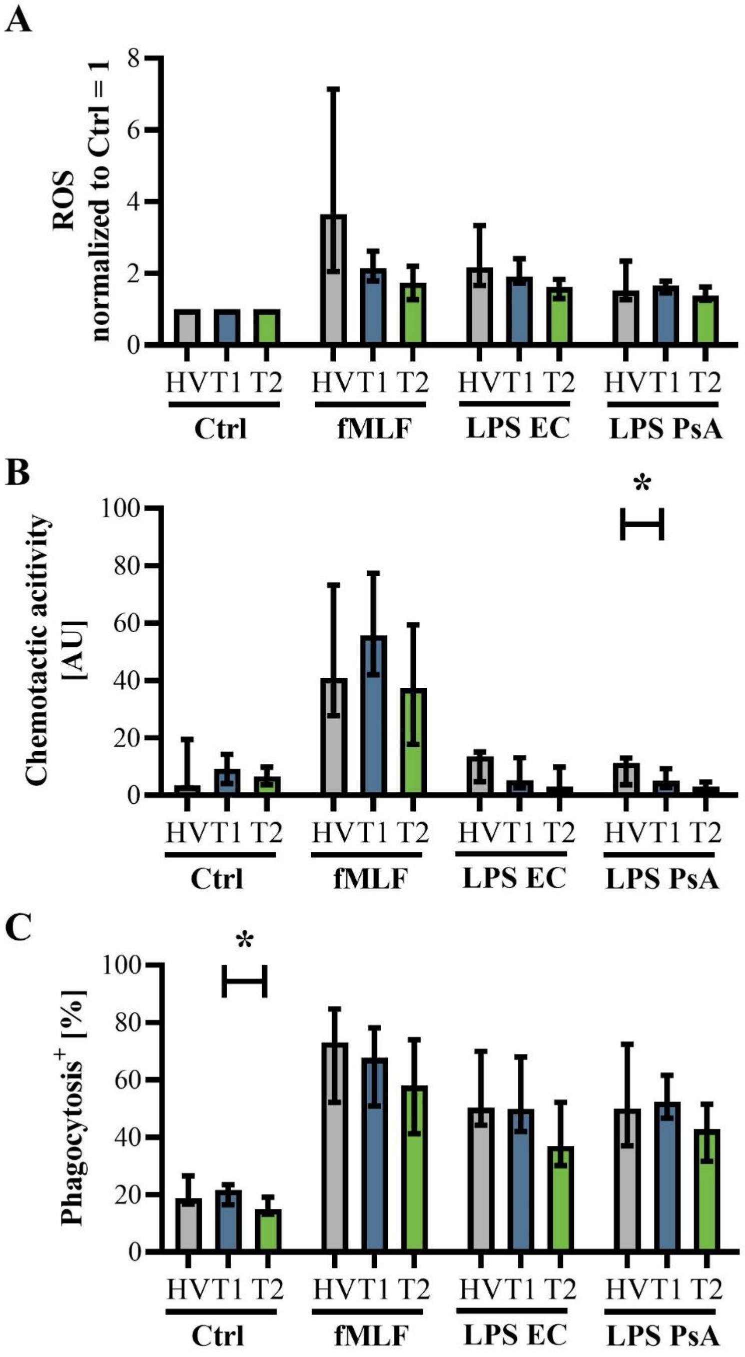

Supplemental Figure 6: Markers of neutrophil function in patients with CF before Elexacaftor–Tezacaftor–Ivacaftor (ETI) treatment (T1) in comparison to age- and sex- matched healthy volunteers (HV) and after 6 months of ETI treatment (T2). **(A)** Generation of radical oxygen species (ROS) after stimulation with 1 µM fMLF, 100 ng/ml LPS from Escherichia coli (LPS EC), or 1 µg/mL LPS from Pseudomonas aeruginosa (LPS PsA) normalized to the respective samples exposed to buffer control, **(B)** chemotactic activity of neutrophils, and **(C)** phagocytic activity. n = 11 – 13, median with interquartile range. * denotes $p < 0.05$.

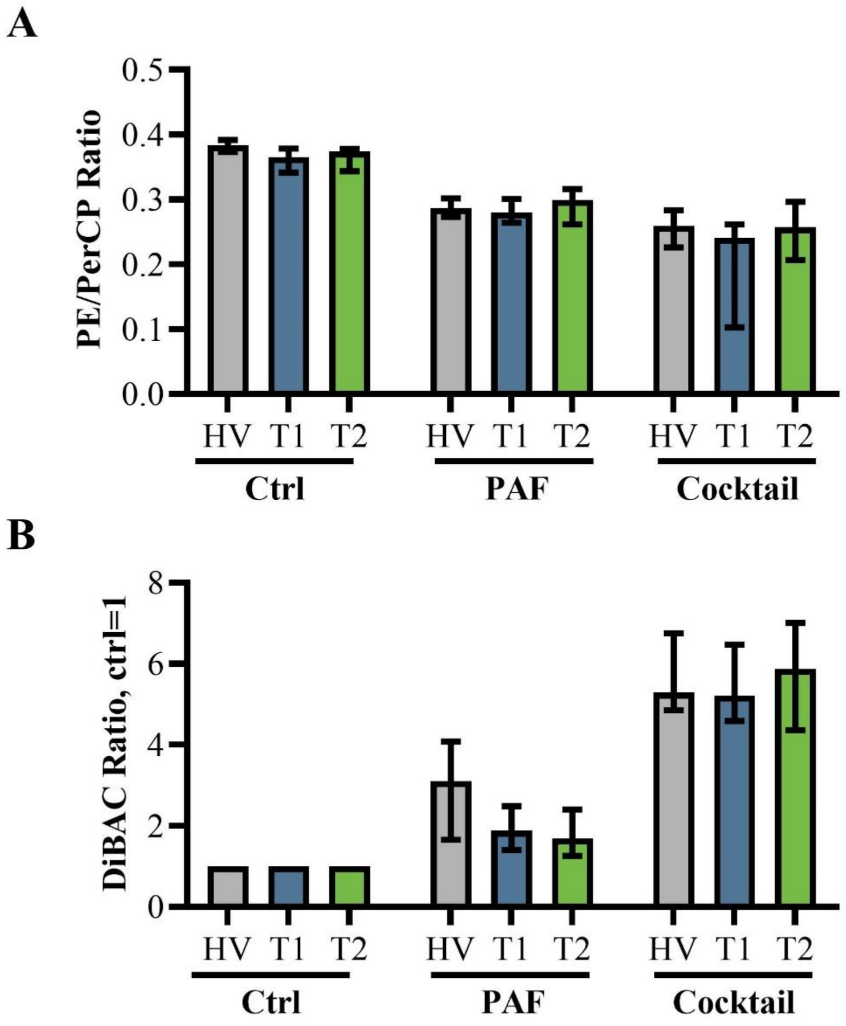

Supplemental Figure 7: Markers of neutrophil cell physiology in patients with CF before Elexacaftor–Tezacaftor–Ivacaftor (ETI) treatment (T1) in comparison to age- and sex- matched healthy volunteers (HV) and after 6 months of ETI treatment (T2). **(A)** Intracellular pH (decrease in PE/PerCP ratio = alkalization) and **(B)** membrane potential (increase in DiBAC fluorescence = depolarization) after stimulation with PAF or a mixture of proinflammatory mediators (Cocktail: 1 µM PAF, 10 µM fMLF, 2.3 µM TNF). n = 11 – 13, median with interquartile range.

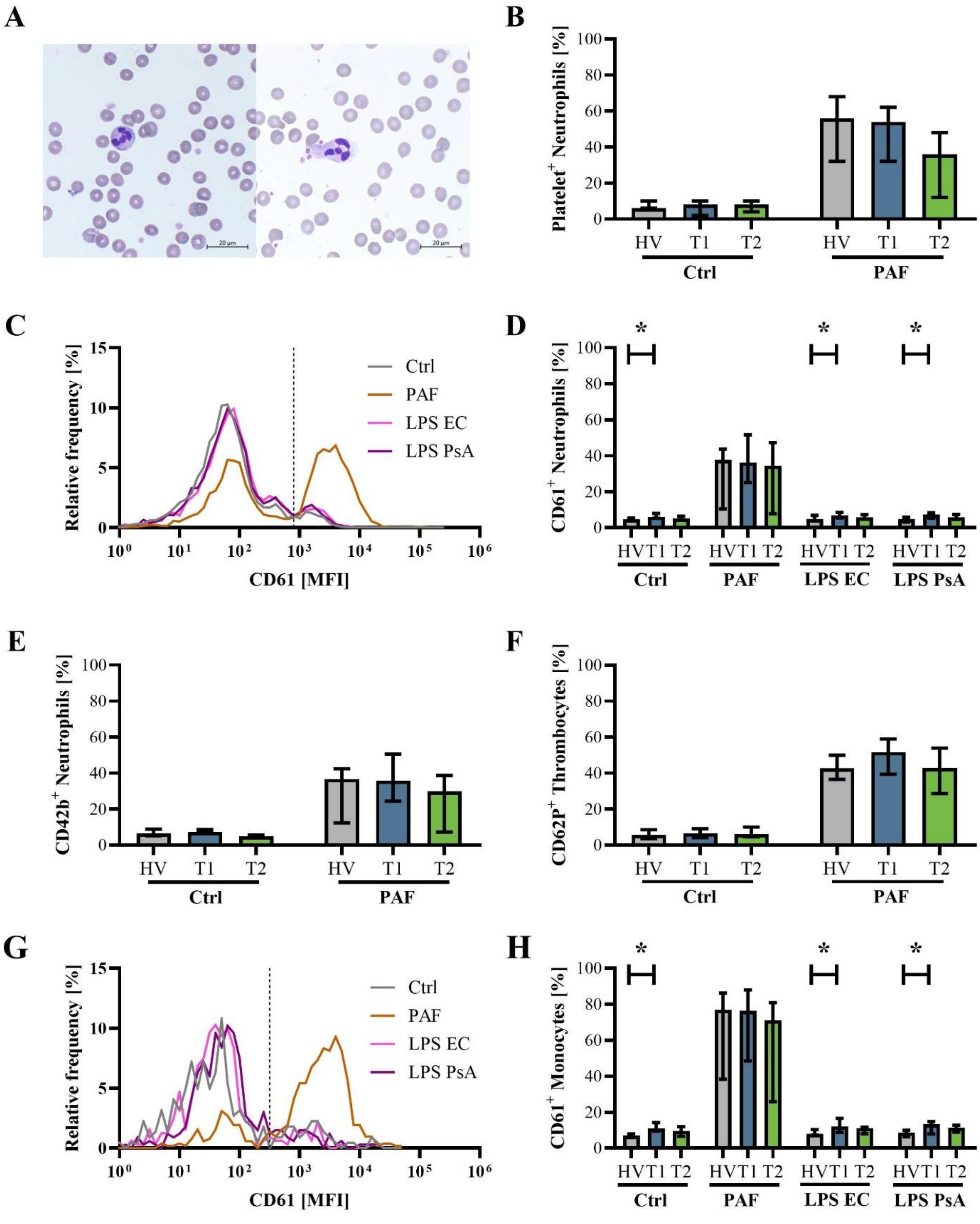

Supplemental Figure 8: Formation of platelet-neutrophil complexes (PNCs) and platelet-monocyte complexes (PMCs) in patients with CF before Elexacaftor–Tezacaftor–Ivacaftor (ETI) treatment (T1) in comparison to age- and sex- matched healthy volunteers (HV) and after 6 months of ETI treatment (T2). The formation of PNCs and PMCs was analyzed in diluted whole blood after 15 minutes of

stimulation with PBS as buffer control or stimulated with 1 µM PAF. **(A)** Representative neutrophil and PNC as detected by light microscopy. **(B)** Evaluation of PNCs by light microscopy. **(C)** Representative histogram of the CD61 signal in neutrophils showing the two populations (neutrophils with or without platelets) in dependence of previous stimulation with PAF (orange), LPS EC (pink), LPS PsA (purple), or buffer control (Ctrl, gray). Analysis of PNC formation by flow cytometry using (**D)** CD61 (as shown in (C)) on neutrophils or **(E)** CD42b on neutrophils as markers of PNCs. **(F)** Analysis of CD62P-expression on thrombocytes as a marker of thrombocyte activation. **(G)** Representative histogram of the CD61 signal in monocytes showing the two populations (monocytes with or without platelets) in dependence of previous stimulation. **(H)** Analysis of PMC formation by flow cytometry as shown in (G) using CD61 on monocytes PAF = platelet-activating factor, LPS EC = lipopolysaccharide from Escherichia coli, LPS PsA = lipopolysaccharide from Pseudomonas aeruginosa. n = 11 – 13, median with interquartile range. * denotes $p < 0.05$.
